# Geometry Effects on Mode I Brittle Fracture in VO-Notched PMMA Specimens

**DOI:** 10.3390/polym13173017

**Published:** 2021-09-06

**Authors:** Robab Bahadori, Majid Reza Ayatollahi, Sergio Cicero, José Alberto Álvarez

**Affiliations:** 1 Fatigue and Fracture Research Laboratory, Center of Excellence in Experimental Solid Mechanics and Dynamics, School of Mechanical Engineering, Iran University of Science and Technology, Narmak, Tehran 16846, Iran; robab_bahadori@yahoo.com; 2 LADICIM, Departamento de Ciencia e Ingeniería del Terreno y de los Materiales, University of Cantabria, Avenida de los Castros, 44, 39005 Santander, Spain; ciceros@unican.es (S.C.); alvareja@unican.es (J.A.Á.)

**Keywords:** VO-notch, PMMA, mode I loading, generalized maximum tangential stress, higher-order terms, crack initiation angle, fracture strength

## Abstract

This paper gathers experimental and theoretical investigations about both the geometry-dependent fracture initiation angle and the fracture strength in VO-notched polymethyl methacrylate (PMMA) specimens under mode I loading conditions. The numerical analyses revealed that despite the application of pure mode I loading on the geometrically symmetric VO-notched samples, the maximum tangential stress occurs at two points symmetrically placed on either side of the notch bisector line. The experimental tests performed on some specimens showed that a crack does not necessarily propagate along the notch bisector line. Stress-based theoretical studies were then carried out to justify the experimental findings. The conventional maximum tangential stress (MTS) criterion gave weak predictions of the fracture. Therefore, the predictions were checked with the generalized MTS (GMTS) criterion by taking into consideration the higher-order stress terms. It was demonstrated that the GMTS criterion predictions have satisfactory consistency with the experimental results of the crack initiation angle and the fracture strength.

## 1. Introduction

Brittle fracture is one of the most frequent failure modes in engineering structures and components made of brittle materials such as polymethyl-methacrylate (PMMA), rocks, ceramics, glasses and alloys at low temperatures, in which fracture is unstable and propagates rapidly. Most failures originate from pre-existing defects or from structural discontinuities such as cracks, flaws, notches, holes, etc. Due to design conditions, the presence of notches in structures and components is unavoidable. Notches act as stress risers and generate an area sensitive to crack initiation around the notch tip, which may lead to catastrophic collapse, especially when dealing with brittle materials. One of the common preventive methods to address this problem in notched components is to make a hole at the notch tip. Consequently, VO-notches may exist in structural components because of the abovementioned repair method on previously existing sharp V-notches. However, similarly to the other types of discontinuities, VO-notches also cause stress concentrations around the (circular) tip, generating a decrease in the load-bearing capacity. Therefore, it is necessary to analyze the brittle fracture of VO-notched components.

Among the different types of notches, V- and U-shaped notches have been profusely analyzed under different loading conditions. Regarding the brittle fracture of notches generated by the stop hole technique on these two types of notches, some research on key-hole notches and VO-notches may be found in [1,2,3], respectively. These works are based on the use of conventional fracture theories to calculate the notch stress intensity factor (NSIF) and consequently the resistance of the notched parts against the applied load. Another issue related to these fracture analyses is the crack initiation angle and the crack growth path. Given that the crack initiation angle and the crack growth path are two of the major factors that define the criticality of the fracture process in engineering structures, researchers have been persuaded to develop tools to predict them [4].

Different theories have been used to assess the fracture of notched components, among which the strain energy density (SED) [5], the maximum tangential stress (MTS) [6] and the averaged SED (ASED) [7] are some well-known examples. Fracture criteria have been established based on the stress field in the vicinity of the notch tip, generally considering (only) the first term of the stress solution [8]. In this regard, for the VO-notches, conventional fracture criteria, such as SED criterion [9,10,11] and the theory of critical distances (TCD) [12,13,14,15], have been widely used in the past. Additionally, the MTS criterion has been successfully used to predict the onset of fracture in various notched specimens, namely VO-notched three-point-bending specimens [9,16] and VO-notched Brazilian disk specimens [10,13].

Due to symmetry in the geometry and loading conditions, one expects that under pure mode I loading, brittle fracture takes place from a crack initiated and extended along the notch bisector line. However, it has been experimentally shown for sharp V-notches that in some cases the crack bifurcates (i.e., nucleates at an angle significantly different from the symmetry line [17]). Similarly, the classical theories of fracture mechanics always assume that cracks initiate from the notch root along the notch bisector line. On the other hand, some recent studies [17,18,19] have substantiated that together with the first term of the stress field, the higher-order terms may be of decisive importance in the brittle fracture analysis of cracked and notched specimens. The impact of the higher-order terms of the stress field around the crack/notch tip on the fracture behavior of components has been physically attributed to geometrical constraints [20,21,22].

On this basis, modified formulations of MTS and SED criteria have recently been employed to predict the crack bifurcation angle and the corresponding fracture load in cracked and notched specimens [17,23,24] under mode I loading by considering the contribution of the higher-order terms of the stress field ahead of the crack/notch tip. The resulting new approaches, i.e., generalized MTS (GMTS) and generalized SED (GSED), are powerful tools for predicting the fracture strength and the crack trajectory. 

Despite the previous analyses with regard to the geometry effects on the fracture behavior of cracked, sharp V-notched and U-notched specimens, there is a paucity in this field for notches with a rounded end. Therefore, the present study investigates the crack bifurcation phenomenon in the VO-notch problem both experimentally and theoretically and, more importantly, predicts the values of bifurcation angle and its corresponding fracture load for a number of VO-notched specimens with different geometry parameters. For this purpose, first, a number of VO-notched specimens with compact tension (CT) and double cantilever beam (DCB) geometries are analyzed under pure mode I loading conditions by means of finite element analysis, obtaining the location of fracture initiation. A series of experimental tests are then performed on CT and DCB specimens made of PMMA, under mode I conditions, to check the consistency between the numerical analyses and the experimental tests. Next, the MTS fracture model is modified for VO-notches by considering the higher-order terms of the analytical stress field. Eventually, the efficiency of the adopted procedures in capturing the geometry effect on the fracture behavior of VO-notched components is assessed and discussed. 

## 2. Numerical Analyses

### 2.1. Analysis Overview

A set of numerical analyses was implemented on three different types of samples containing VO-shaped notches, determining the corresponding variation in tangential stresses (TS) around the notch tip. The goal of the numerical analyses is to check the impact of various geometry parameters (e.g., width/height ratio, notch opening angle, notch root radius) on the TS distribution around the VO-notch border. These numerical studies provide an objective assessment of how the TS contours are distributed, and about the location at which the maximum TS takes place.

**Figure 1:**
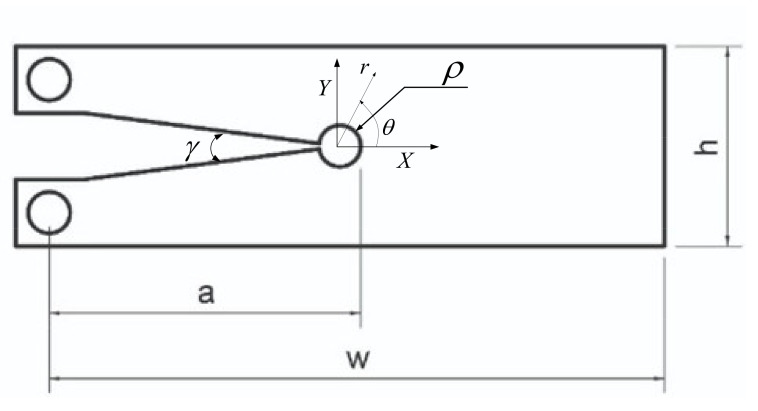
Schematic and main geometrical parameters of the different specimens.

Figure 1 shows the geometry and the dimensions of the VO-notched specimen models, and also the components of the polar coordinate system. The height (h) of all specimens was fixed at 30 mm. The values of the width, W, and the notch depth, a, for each of the three particular geometries (CT, DCB^1^ and DCB^2^) are shown in Table 1. Additionally, four different notch radii (ρ = 0.0 mm, 0.125 mm, 1.0 mm, 1.5 mm and 2.5 mm) and two different notch open angles (γ = 15°, 30°) are analyzed. It should be noted that a VO-notch with a notch tip radius of 0.0 mm represents a sharp V-notch. In all specimens, the notch depth variation is proportional to the width variation so that the ratio of a to W remains constant at a/W = 0.5.

**Figure 2:**
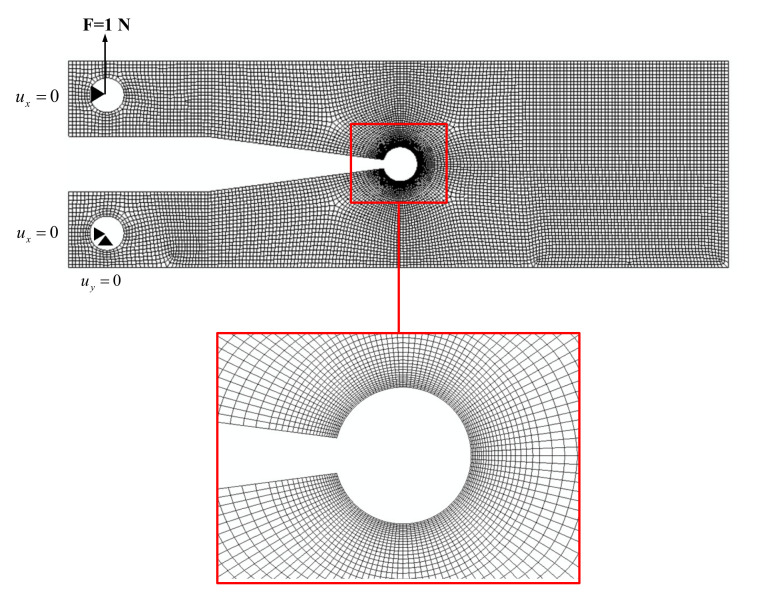
FE model and mesh pattern for DCB^1^ specimen near the VO-notch tip (ρ = 1 mm, γ = 15°).

Numerical analyses were performed using the commercial finite element (FE) code ABAQUS (Dassault Systèmes Simulia Corp., Johnston, RI, USA), employing isoparametric 8-node biquadratic plane stress quadrilateral elements. A mesh-sensitivity analysis was performed to ensure the reliability of the outputs, concluding that a mesh size of 0.1 mm for the region in the vicinity of the notch tip provided reliable results. It was found that a total number of about 6500 elements for the CT specimen, 13,000 elements for the DCB^1^ specimen and 19,000 elements for the DCB^2^ specimen resulted in an appropriate convergence. Figure 2 shows the mesh grid and the boundary conditions of the DCB^1^ model with a notch root radius of 1 mm and notch opening angle of 15°.

### 2.2. Bouandary Conditions 

Mode I loading in the test specimens was simulated according to the boundary and loading conditions shown in Figure 2. Tensile load (F) was applied to the upper hole of the specimen and the lower hole was fixed against the vertical and horizontal directions. All the models were analyzed under the hypothesis of linear elastic behavior, pure mode I loading conditions and at the reference applied load (F) of 1 N. The elastic modulus (E) and the Poisson’s ratio (υ) of the material were selected as 2900 MPa and 0.38, respectively, according to the reported values for this PMMA in [25]. The thickness (t) of the specimens was set at 5 mm, as this value was used in the experimental program.

### 2.3. Determination of the Maximum TS Direction

**Figure 3:**
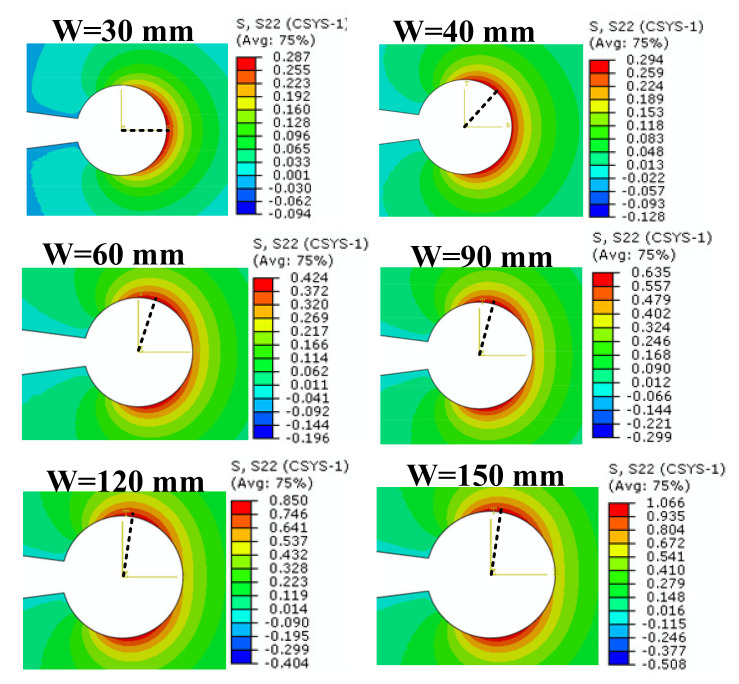
TS contours around VO-notches for FE models with various widths (ρ = 2.5 mm, γ = 15°).

The numerical studies began evaluating the impact of the width on the TS contour distribution around the VO-notch border. Next, the evaluation continued with the effect of the different notch root radii. Figure 3 shows the TS contours around the VO-notched models with different widths of W = 30, 40, 60, 90, 120 and 150 mm, while the notch root radius and the notch opening angle were fixed at 2.5 mm and 15°, respectively. For all cases, the direction of the maximum TS is indicated with a dashed line. As shown in Figure 3, for the model with W = 30 mm (CT configuration), the stress contours around the notch edge have one single lobe whose convexity is located exactly in front of the notch tip and is symmetric with respect to the notch bisector line. By increasing the width of the specimens, the TS contours deviate in two lobes located at both sides of the notch bisector line. It can be seen from Figure 3 that despite the symmetry in both geometry and loading conditions, the maximum TS for the DCB models takes place along an angular direction that is significantly different from the notch bisector line.

**Figure 4:**
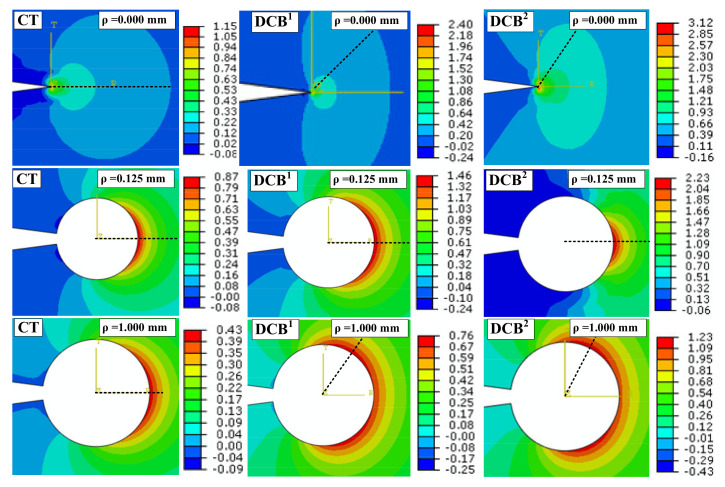
TS contours around VO-notches for CT and DCB models with various radii (γ = 15°).

Figure 4 shows the TS contours around the notch edge for the VO-notched CT, DCB^1^ and DCB^2^ models with notch radii of 0.0, 0.125 and 1.0 mm while the notch opening angle is fixed at 15°. The analyses show that the roundness at the V-notch root is a crucial geometrical parameter in deviation of the maximum TS direction from the notch bisector line. It can be observed that for both CT and DCB models with ρ = 0.125 mm, the crescent shape of the TS contours is located exactly in front of the notch tip, so that the symmetry line coincides with the notch bisector line. In DCB models with ρ = 0.00 mm and ρ = 1.00 mm, the crescent shape disappears and the TS contours form in two lobes on both sides of the notch bisector line.

**Figure 5:**
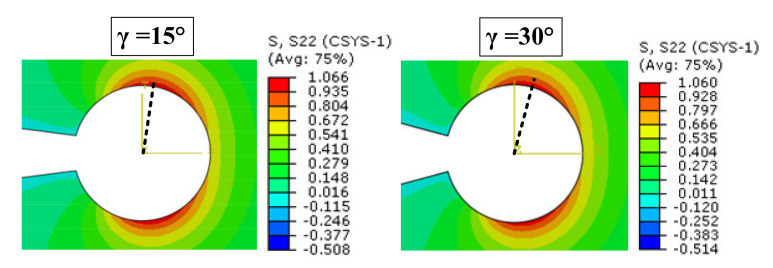
TS contours around VO-notches for DCB^2^ model with various notch opening angles (ρ = 2.5 mm).

Figure 5 illustrates the variations in TS with respect to notch opening angle for a DCB^2^ model with notch root radius of ρ = 2.5 mm. It is derived from Figure 3, Figure 4 and Figure 5 that the values of maximum TS increase by increasing the width or by decreasing the notch root radius or by decreasing the notch opening angle. 

## 3. Experimental Validation

Polymethylmetacrylate (PMMA) is a well-known test material which has been extensively used in the past for investigating brittle fracture behavior of engineering materials. The experimental validation was performed here using PMMA specimens, with brittle behavior and homogeneous isotropic structure. The material properties of PMMA are reported in [25]: (Young’s modulus) E = 2900 MPa, (Poisson’s ratio) υ = 0.38, (tensile strength) σ_t_ = 26.5 MPa and (fracture toughness) K_Ic_ = 39.07 MPa·m^0.5^. 

For the experimental mode I fracture tests, the CT, DCB^1^ and DCB^2^ geometries (previously analyzed in the numerical solutions) were selected. The fabricated specimens were obtained from a PMMA sheet with a thickness of 5 mm, and were cut by means of a CNC laser cutter machine. The cutter machine has a precision of about 0.25 mm. The specimen widths (W) were 30, 90 and 150 mm, and a/W was kept constant at 0.5. In this part of the experiment, the values of the notch tip radii were 2.5 mm and 3.0 mm.

**Figure 6:**
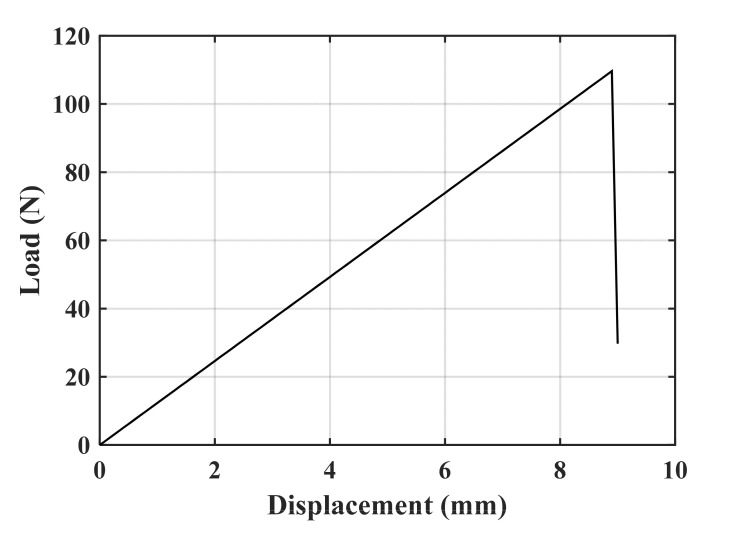
Example of load–displacement curve. VO-notched CT specimen radii (ρ = 2.5 mm; γ = 30°).

**Figure 7:**
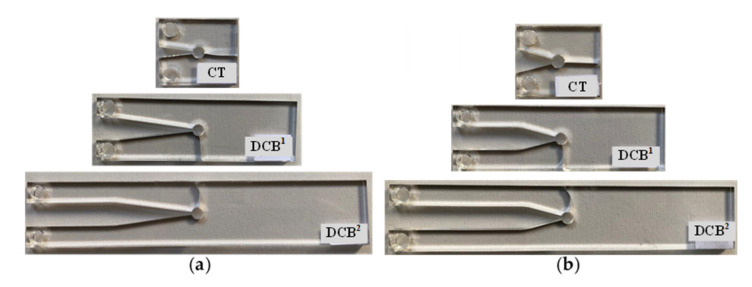
CT, DCB^1^, and DCB^2^ VO-notched specimens fractured under mode I loading condition. Ρ = 2.5 mm. (**a**) γ = 15°; (**b**) γ = 30°.

A tensile mode I load was applied on the test specimens at room temperature and at a constant rate of 0.5 mm/min. The repeatability of the results was checked by doing tests on the three specimens per geometry. In all the tests, it was observed that, after crack initiation, the crack growth in the PMMA specimens was quick and unstable. Figure 6 illustrates the load–displacement curve of a CT specimen with a notch root radius of 2.5 mm and a notch opening angle of 30°. It can be observed that the load–displacement curve is completely linear with a sharp load drop at the onset of fracture. Therefore, the linear elastic fracture mechanics (LEFM) assumption near the notch tip is reasonable and the specimens can be considered to have failed by brittle mechanism of fracture. Figure 7 shows two groups of the broken CT, DCB^1^ and DCB^2^ specimens with the notch root radius of 2.5 mm. It can be seen that, unlike the CT specimens, for the DCB specimens, cracks did not initiate in front of notch tip and propagated in a curvilinear path, not along the notch bisector line. Table 2 shows the measured fracture loads (F_i_, I = 1, 2, 3) for each configuration in the experimental tests (3 per condition), together with the experimental fracture angles (θ_0,i_, I = 1, 2, 3). 

## 4. Mode I Brittle Fracture Criterion

### 4.1. Theoretical Reasoning

The first question that is raised when performing theoretical studies of the brittle fracture of notched specimens is the definition of the stress field around the notch tip, which is the basis for the subsequent definition of an appropriate fracture criterion. The elastic stress fields around VO-notches were proposed in [26], as series of n terms. In this section, these stress solutions are used with a well-known fracture model, the MTS criterion, to establish a modified fracture criterion.

**Figure 8:**
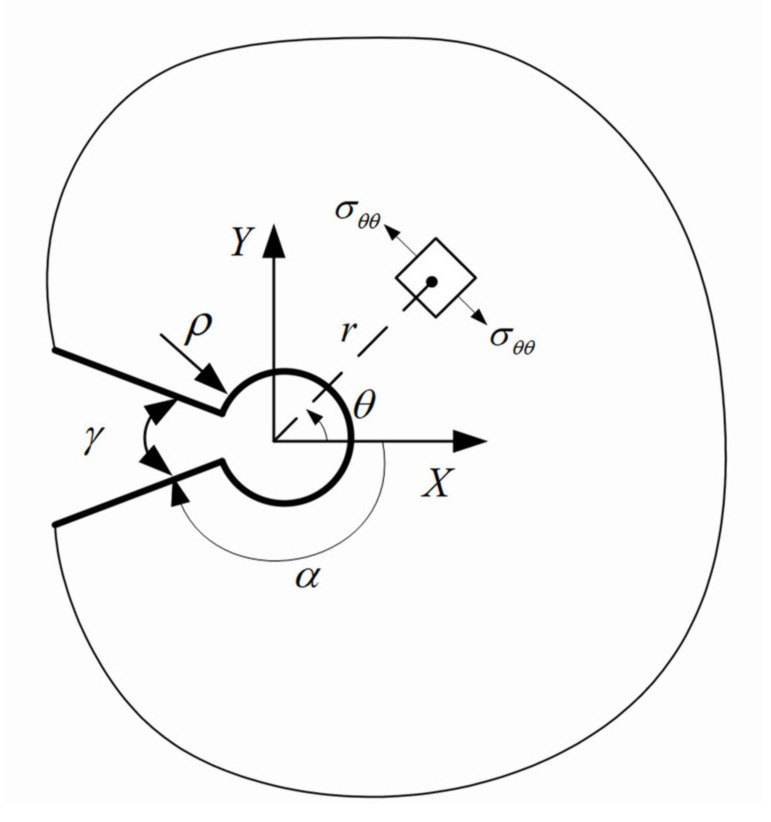
Geometrical parameters of a VO-notch, and TS component in both the polar and Cartesian coordinates system.

Figure 8 shows a schematic VO-notch and the normal stress component (σ_θθ_) in the polar coordinate system. In this figure, γ stands for the notch opening angle and α can be obtained from the notch angle as 2α = 2π-γ.

Under mode I load, the TS component around a VO-notch in polar coordinates (Figure 8) can be expressed as follows [26]:

(1) σθθ=∑n=1∞ReAnrλn−1cos1−λnθ1+λn+ρr2λnψn1θ+ρr2λn+1ψn2θχn1θ+ϕncos1+λnθ1+ρr2λn1−λn+ρr2λn+22+λn

where Re stands for the real part or the different terms, A_n_ are coefficients depending on the analyzed geometry, n is the order of term in the stress series expansion and the auxiliary parameters ϕ_n_, ψ_n1_, ψ_n2_ and χ_n1_ are as follows:

(2) $$\phi_{n}=-\left(1-\lambda_{n}\right)\frac{\sin\left(1-\lambda_{n}\right)\alpha }{\sin\left(1+\lambda_{n}\right)\alpha }$$

(3) $$\psi_{n1}\left(\theta \right)=\frac{2\sin\left(\lambda_{n}\right)\theta \cos\left(\lambda_{n}-1\right)\theta +\left(1-\lambda_{n}\right)\sin\left(2\lambda_{n}-1\right)\theta }{\sin\theta }$$

(4) $$\psi_{n2}\left(\theta \right)=\frac{\left(\lambda_{n}-2\right)\sin2\left(1-\lambda_{n}\right)\theta }{\sin\theta }$$

(5) $$\chi_{n1}\left(\theta \right)=\frac{\cos\left(\lambda_{n}\right)\theta }{\cos\left(\lambda_{n}-1\right)\theta }$$

with λ_n_ being the mode I eigenvalues that can be calculated through the following characteristic equations:

(6) $$\lambda_{n}\sin\left(2\alpha \right)+\sin\left(2\lambda_{n}\alpha \right)=0$$

Solving Equation (6) leads to the real and complex roots of n eigenvalues.

It has been substantiated that the normal stress plays a crucial role in the fracture of brittle materials [27,28]. The MTS theory is one of the most widely used models for the analysis of brittle fracture processes. According to the MTS criterion, brittle fracture near a VO-notch takes place normal to the direction of the maximum TS, θ_0_, when the TS at a critical distance of r_cr_ from the notch tip reaches its critical value, (σ_θθ_)_crit_. The MTS criterion is mathematically expressed as:

(7) $${\left.\frac{\partial \sigma_{\theta \theta }}{\partial \theta }\right)}_{\theta =\theta_{0}}=0,\;{\left.\frac{\partial^{2}\sigma_{\theta \theta }}{\partial \theta^{2}}\right)}_{\theta =\theta_{0}}<0$$

(8) $$\sigma_{\theta \theta }\left(r_{cr},\theta_{0}\right)={\left(\sigma_{\theta \theta }\right)}_{crit}$$

An appropriate critical distance from the notch tip corresponding to the MTS criterion has been proposed by Sapora et al. [28] as:

(9) $$r_{cr}=\frac{1}{2\pi }{\left(\frac{K_{Ic}}{{\left(\sigma_{\theta \theta }\right)}_{crit}}\right)}^{2}$$

where K_Ic_ is the material plane strain fracture toughness.

For brittle materials, the critical stress (σ_θθ_)_crit_ can be considered as the conventional ultimate tensile strength σ_t_ [29]. By using the values of K_Ic_ and σ_t_ presented in Section 3, the critical distance can be simply obtained as r_cr_ = 0.35 mm. 

The relation of the mode I VO-notch stress intensity factor (NSIF) $K_{I}^{\mathrm{VO}}$ with the first coefficient of the stress field A_1_ at notch tip [30] could be written as follows:

(10) $$\begin{array}{l} K_{I}^{VO}=\sqrt{2\pi }r^{1-\lambda_{1}}\sigma_{\theta \theta }\left(r=\rho ,\theta =\theta_{0}\right)\\ =\sqrt{2\pi }\left[\left(1+\lambda_{1}\right)+\psi_{11}\left(\theta_{0}\right)+\psi_{12}\left(\theta_{0}\right)\chi_{11}\left(\theta_{0}\right)+4\phi_{1}\right]A_{1} \end{array}$$

Now, considering the conventional MTS criterion, under mode I loading conditions, the crack initiates in front of the notch tip and propagates along the notch bisector line. Applying the crack propagation condition (Equations (7) and (8)) to the TS general expression (Equation (1)), based on the use of higher-order terms of the stress field, leads to the generalized MTS (GMTS) criterion for VO-notches. The TS defined by Equation (1) can be rewritten as a series of terms by taking into account the higher-order terms in addition to the NSIF:

(11) $$\sigma_{\theta \theta }=\frac{K_{I}^{VO}}{\sqrt{2\pi }\left[\left(1+\lambda_{1}\right)+\psi_{11}\left(0\right)+\psi_{12}\left(0\right)\chi_{11}\left(0\right)+4\phi_{1}\right]}M_{1}\left(r,\theta \right)\;+\overset{\infty }{\underset{n=2}{\sum }}\left\{A_{n}M_{n}\left(r,\theta \right)\right\}$$

where:

(12) $$\begin{array}{l} M_{n}\left(r,\theta \right)=r^{\lambda_{n}-1}\left\{\cos\left(1-\lambda_{n}\right)\theta \left[\left(1+\lambda_{n}\right)+{\left(\frac{\rho }{r}\right)}^{2\lambda_{n}}\psi_{n1}\left(\theta \right)+{\left(\frac{\rho }{r}\right)}^{2\lambda_{n}+1}\psi_{n2}\left(\theta \right)\chi_{n1}\left(\theta \right)\right]\right.\\ \left.+\phi_{n}\cos\left(1+\lambda_{n}\right)\theta \left[1+{\left(\frac{\rho }{r}\right)}^{2\lambda_{n}}\left(1-\lambda_{n}\right)+{\left(\frac{\rho }{r}\right)}^{2\lambda_{n}+2}\left(2+\lambda_{n}\right)\right]\right\} \end{array}$$

The fracture initiation location when applying the GMTS criterion can be achieved by first-order derivative of Equation (11):

(13) $${\left.\frac{\partial \sigma_{\theta \theta }}{\partial \theta }\right)}_{\theta =\theta_{0}}=\frac{K_{I}^{VO}}{\sqrt{2\pi }\left[\left(1+\lambda_{1}\right)+\psi_{11}\left(0\right)+\psi_{12}\left(0\right)\chi_{11}\left(0\right)+4\phi_{1}\right]}\frac{\partial M_{1}\left(r,\theta \right)}{\partial \theta }\;+\overset{\infty }{\underset{n=2}{\sum }}\left\{A_{n}\frac{\partial M_{n}\left(r,\theta \right)}{\partial \theta }\right\}=0$$

### 4.2. Fracture Strength Evaluation

In this part of the research, the fracture strength of VO-notched components was studied by considering the GMTS criterion, which has been described in the previous subsection. By substituting the obtained fracture angle from Equation (13) into Equation (11), the fracture strength $K_{\mathrm{If}}^{\mathrm{VO}}$ at the critical distance from the notch tip can be calculated by using the following relation:

(14) $$\begin{array}{l} {\left(\sigma_{\theta \theta }\right)}_{cr}=\frac{K_{If}^{VO}}{\sqrt{2\pi }\left[\left(1+\lambda_{1}\right)+\psi_{11}\left(0\right)+\psi_{12}\left(0\right)\chi_{11}\left(0\right)+4\phi_{1}\right]}M_{1}\left(\rho +r_{cr},\theta_{0}\right)\\ +\overset{\infty }{\underset{n=2}{\sum }}\left\{A_{n}M_{n}\left(\rho +r_{cr},\theta_{0}\right)\right\} \end{array}$$

where $K_{\mathrm{If}}^{\mathrm{VO}}$ represents the VO-notch fracture strength developed at the fracture load onset.

By considering the conventional MTS, which always estimates that the crack initiation angle under pure mode I is zero, θ_0_ = 0, and is only focused on the first term of the series, the critical TS takes the following form:

(15) $${\left(\sigma_{\theta \theta }\right)}_{cr}=\frac{K_{Ic}^{VO}}{\sqrt{2\pi }\left[\left(1+\lambda_{1}\right)+\psi_{11}\left(0\right)+\psi_{12}\left(0\right)\chi_{11}\left(0\right)+4\phi_{1}\right]}M_{1}\left(\rho +r_{cr},0\right)$$

where $K_{\mathrm{Ic}}^{\mathrm{VO}}$ is the VO-notch fracture toughness. Equating the last two relations (Equations (14) and (15)) yields the relationship for the fracture strength ratio, which is defined as the proportion of the fracture strength to the conventional VO-notch fracture toughness:

(16) $$\frac{K_{If}^{VO}}{K_{Ic}^{VO}}=\frac{M_{1}\left(\rho +r_{cr},0\right)}{M_{1}\left(\rho +r_{cr},\theta_{0}\right)+\sum_{n=2}^{\infty }\left\{\frac{A_{n}}{A_{1}}M_{n}\left(\rho +r_{cr},\theta_{0}\right)\right\}}$$

Equation (16) shows that the higher-order terms may have a strong effect on the fracture strength.

## 5. Results and Discussion

### 5.1. Stress Field Coefficients and Tangential Stress Distributions

In order to evaluate the results, the coefficients of the notch tip asymptotic stress field under mode I loading have been (firstly) determined through the finite element over-deterministic (FEOD) method. The FEOD method has been proposed to calculate the first n number of coefficients of the stress field, A_n_, for sharp V-notches, and has recently and successfully been implemented for rounded V-notches [26]. In the second step, the accuracy of the truncated TS series solution, considering the first three terms on the basis of the calculated coefficients, was compared with the TS distribution obtained from the numerical analysis. Finally, the experimental results have been compared with the theoretical estimations obtained from the MTS and GMTS criteria.

Thus, the FEOD method was used to calculate the coefficients of the stress series. Table 3 shows the first three coefficients of the stress series in the vicinity of the VO-notch tip obtained under a reference applied load of F = 1 N.

**Figure 9:**
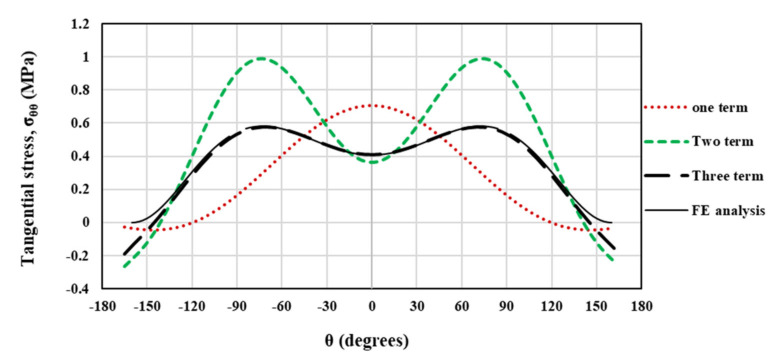
TS distribution provided by FE results and (truncated) theoretical solutions for VO-notched DCB^1^ specimen with the root radius of 2.5 mm and opening angle 30°.

Moreover, this subsection also provides a comparison between theoretical and numerical TS distributions around the VO-notch tip obtained from the truncated stress series predictions and the FE simulations. To this end, the coefficients reported in Table 3 are used to establish the truncated stress series. Figure 9 illustrates the accuracy of the theoretical results obtained from the truncated TS series distribution when considering the first term, the first two terms and first three terms. The TS distributions in this figure are determined at the critical distance ahead of the VO-notch edge in the DCB specimen with the width (W) being 90 mm, the notch root radius being 2.5 mm, and notch opening angle being 30°. As is obvious in Figure 9, the stress series distribution generated when considering only the first term provides a maximum TS in the angular direction of θ = 0° (bisector line). However, when considering the first two and the first three terms, the maximum TS no longer remains along θ = 0° (i.e., the fracture angle does not coincide with the notch bisector line). In addition, by considering the first three terms in the asymptotic solution of the stress field, the theoretical predictions coincide with the FE numerical solution. Additionally, the angular deviation of the maximum TS from the θ = 0° direction, which is seen in Figure 3, Figure 4 and Figure 5, can be described by considering the higher-order terms in the stress field solution. Consequently, when applying the GMTS criterion to VO-notches, considering the first three terms in the TS series (i.e., considering the geometry effects) provides more accurate results. 

### 5.2. Crack Initiation Angular Direction

**Figure 10:**
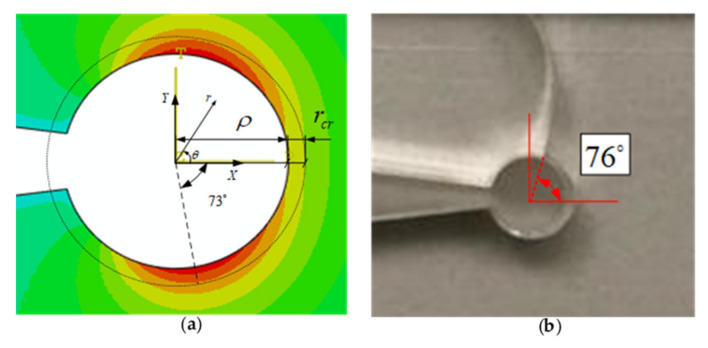
Crack initiation angle for DCB^2^ specimen with notch radius 2.5 mm and opening angle 15°. (**a**) The finite element analysis; (**b**) experimental result.

Figure 10 compares one simulated (FE) sample with the corresponding experimental test. Figure 10a shows the angle of the maximum TS (θ_0_) at a critical distance (r_cr_) of 0.35 mm from the notch tip and obtained from FE analysis, while Figure 10b shows the angular direction of crack initiation in the experimental test for the VO-notched DCB^2^ specimen with the notch opening angle of 15° and the notch root radius of 2.5 mm. It can be observed that the TS at the critical distance has reached its maximum value at the angle of 73° under pure mode I loading, which is consistent with the experimental fracture initiation angle of 76°. Moreover (see Figure 10a), the maximum TS points are formed in two lobes symmetrically located on both sides of the notch bisector line. In Figure 10b, although the specimen is subjected to mode I loading, the crack does not initiate at the notch bisector line and does not propagate along this line either. Moreover, the crack is kinked out to the sideward edge of the specimen. 

**Figure 11:**
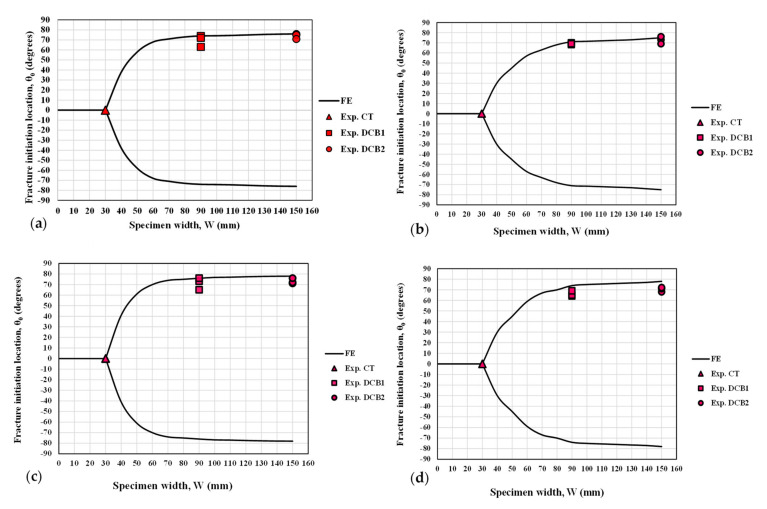
Crack initiation location predicted via GMTS and comparison with the corresponding experimental results for the CT, DCB^1^ and DCB^2^ specimens: (**a**) γ = 15°, ρ = 2.5 mm; (**b**) γ = 15°, ρ = 3.0 mm; (**c**) γ = 30°, ρ = 2.5 mm; (**d**) γ = 30°, ρ = 3.0 mm.

Figure 11 graphically compares the crack initiation angle obtained from the analytical criterion (GMTS) with the experimental measurements obtained in the broken specimens, in terms of W (the specimen width) and under mode I loading. An alternative report of the experimental and theoretical values of the crack initiation angle is shown in Table 4. It is obvious that the fracture angular location in the CT specimens always remains along the notch bisector line, θ = 0°. As W increases, the fracture angle increases so that the DCB^2^ specimens undergo the fracture at larger angles in comparison with the DCB^1^ specimens. The small discrepancies are here attributed to the non-ideal conditions of the experimental tests and the material structure, while theory considers ideal conditions. It is important to note that the crack can deviate equally either in the positive or negative angular direction, depending on any possible minor triggering parameter. The DCB specimens shown in Figure 7 are good examples for this observation. Accordingly, the average values of absolute fracture angles are shown in Table 4.

In the next subsection, the impact of the roundness of the V-notch tip is assessed.

### 5.3. Additional Analysis on Blunt V-Notches

**Figure 12:**
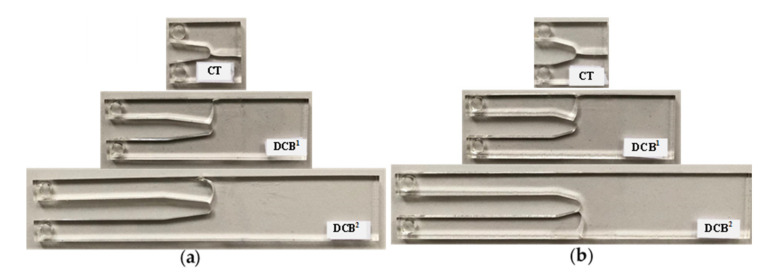
CT, DCB^1^ and DCB^2^ blunt V-notched specimens fractured under mode I loading condition: (**a**) γ = 15°, ρ = 2.5 mm; (**b**) γ = 30°, ρ = 3.0 mm.

In order to assess the impact of the notch tip roundness on the fracture angle of the V-notched specimens, some numerical and experimental studies were carried out on the CT, DCB^1^ and DCB^2^ specimens containing blunt V-notches. Figure 12 illustrates blunt V-notched specimens fractured under mode I loading, while Table 5 shows the measured fracture loads and the angular fracture initiation location of the compared blunt V-notched specimens, comparing such values with those previously obtained in VO-notched specimens. It can be observed (Table 5) that for both types of notches, CT specimens fractured and cracks propagated along the notch bisector line, while for the DCB specimens, the crack initiates from a given angular location with respect to the notch bisector line. On the other hand, the VO-notched DCB specimens have a larger angular crack initiation location than the blunt V-notched DCB specimens. In addition, the fracture loads related to the blunt V-notched specimens are generally higher than those developed by the VO-notched ones.

The impact of the minimum roundness at the V-notch root (i.e., the minimum achievable size by a laser cutting machine with the precision of 0.25 mm which produces a circle with the diameter of 0.25 mm at notch tip) on the crack initiation angle was also checked. It is shown in Figure 4 that for VO-notched DCB samples with a notch root radius of zero (sharp V-notches), the initiated crack at the notch tip will immediately kink from the notch bisector line, while for a root radius of 0.125 mm, the crack will propagate along the notch bisector line. For broader notch root radii, the crack initiation angle takes larger values. Table 6 shows a comparison between the experimental results obtained from the sharp V-notched and from the VO-notched (ρ = 2.5 mm) specimens. It is seen from this table that when a sharp notch turns into a VO-notch, both the fracture load and the fracture initiation angle increase (except for the case of CT specimens). This holds true for notch opening angles of γ = 15° and 30°.

**Figure 13:**
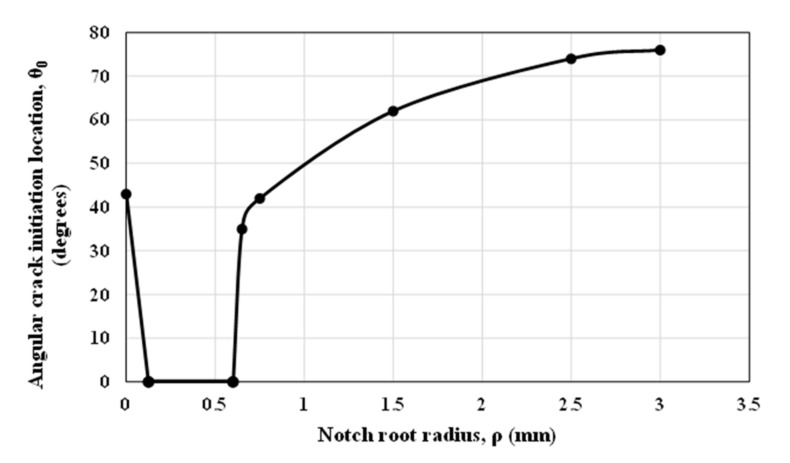
Crack initiation angle variations obtained from numerical analyses for different notch root radii in DCB^1^ blunt V-notched specimens (γ = 15°).

A similar numerical analysis was performed on blunt V-notched specimens. Figure 13 compares the crack initiation angle in terms of the notch root radius for the DCB^1^ specimens containing a V-notch with the notch opening angle of 15°.

**Figure 14:**
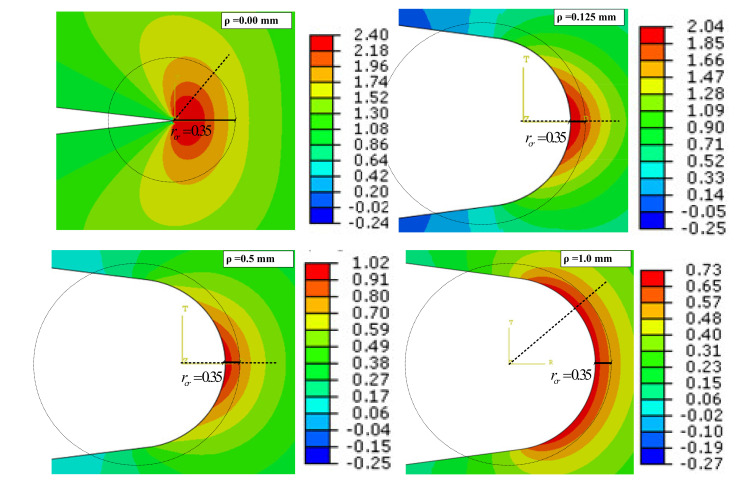
Numerical TS variations around blunt V-notch edge in DCB^1^ specimens with different notch root radii (γ = 15°).

As shown in Figure 13, for a sharp V-notch (in which ρ = 0.0 mm) with an opening angle of 15° under mode I loading, the crack does not initiate along the notch bisector line [17] (the fracture angular location is larger than 40°), while the introduction of a fine roundness at the root of the V-notch causes the crack to initiate along the notch bisector line (θ = 0°). The crack deviates again from the bisector line when the notch radius is larger than 0.6 mm. To study this behavior in more detail, a number of numerical analyses were performed on blunt V-notched specimens with various notch root radii. Figure 14 illustrates the contours of TS and the critical distance from the blunt V-notch border. 

It is seen from Figure 14 that when ρ = 0.0 mm and 1.0 mm, the maximum TS occurs at an angular location about 40°, while for ρ = 0.125 mm and 0.5 mm it takes place at the angle θ = 0° (i.e., along the notch bisector line). The change in the angle of maximum TS could be attributed to the evolution of notch tip singularity when a sharp notch turns into a blunt notch and when the root radius increases. This can be observed from the shapes of TS contours shown in Figure 14.

**Figure 15:**
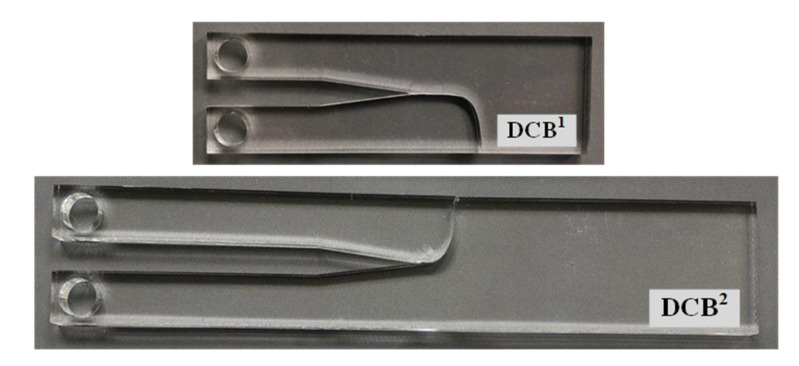
Broken blunt V-notched DCB^1^ and DCB^2^ specimens under mode I loading. γ = 15°; ρ = 0.125 mm.

Figure 15 shows the broken specimens containing a blunt V-notch. As expected, in the blunt V-notched DCB specimens with a notch radius of 0.125 mm and opening angle of 15°, the crack is initiated in front of the notch tip, along the notch bisector line. After the crack initiates with an angular direction of 0° and certain propagation along the notch bisector line, it finally propagates following a curvilinear path [31].

### 5.4. Fracture Strength 

The theoretical fracture strength, $K_{\mathrm{If}}^{\mathrm{VO}}$, can be calculated for each specimen by using Equation (16). For this purpose, the GMTS criterion is applied, taking into consideration the first three terms of the stress series solution. On the other hand, the experimental fracture strengths are obtained by inserting the experimental fracture loads into the FE models. Then, the TS obtained from the FE analysis on the VO-notch border and along the experimentally determined fracture angle θ_0_ direction is entered into Equation (10), with the resulting $K_{I}^{\mathrm{VO}}$ being considered as the experimental $K_{\mathrm{If}}^{\mathrm{VO}}$.

**Figure 16:**
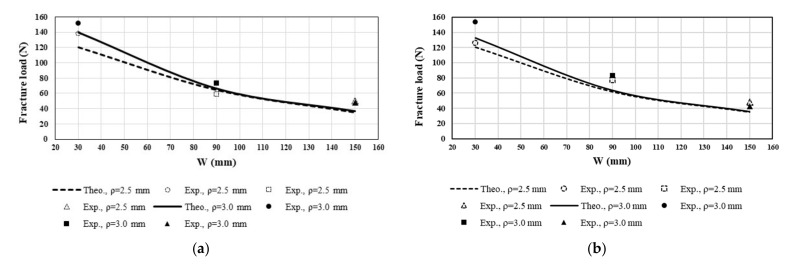
Experimental and theoretical (GMTS) fracture load variation in terms of specimen width (W) for VO-notched CT and DCB specimens. (**a**) γ = 15°; (**b**) γ = 30°.

Figure 16 shows a comparison between the theoretical fracture loads and the experimental results. According to the results shown in the figure, the fracture load increases by decreasing either the notch opening angle or the width of the specimen, and increases by raising the notch root radius. The following procedure was adopted to calculate the theoretical fracture load. First, by applying a reference load of 1 N under mode I loading conditions, the first three coefficients of the theoretical stress series were obtained for each specimen through the FEOD approach. Then, the GMTS theory was established on the basis of the first three terms. According to Equations (7) and (8), the maximum TS can be determined at the critical distance from the notch border and along the direction of θ_0_. The theoretical fracture load is an applied load under which the maximum TS reaches the material tensile strength, σ_t_ [29]. Considering the LEFM assumptions, the maximum TS obtained from the GMTS criterion under a reference load of F = 1 N has a linear relation with the ultimate strength of the material. Therefore, the fracture load can be calculated by multiplying the reference load (i.e., 1 N) by the ratio ultimate tensile strength to GMTS maximum TS (for F = 1N).

Table 7 reports the calculated values of the theoretical and experimental fracture strengths. The calculated theoretical fracture strength includes the values obtained through both the MTS and GMTS criteria. It can be seen from Table 7 that, on the one hand, there is a noticeable difference between the GMTS and MTS criteria estimations for $K_{\mathrm{If}}^{\mathrm{VO}}$ and, on the other hand, there is a good agreement between the experimental results and the theoretical GMTS predictions for the fracture strength of the VO-notched samples. Furthermore, the fracture strength of the DCB specimens noticeably differs from that observed in the CT specimens. These differences may be attributed to the different geometries of the specimens, since the fracture strength is a function of the higher order terms of the stress field (Equation (16)). According to Table 7, for a constant notch opening angle (γ), by decreasing the notch root radius (ρ) or by increasing the width of the specimens (W), the fracture strength decreases. 

Some notes are added here to underline the scientific importance of the results presented in this paper. It was shown that in some practical cases, a VO-notched specimen subjected to symmetric loading experiences crack initiation at an angle significantly different from the notch bisector line. Due to symmetry in both geometry and loading conditions, one expects that the crack always initiates from the boundary of VO-notch along the symmetry (or bisector) line. Depending on the geometry parameters of the VO-notched specimen, the crack bifurcation out of the symmetry line (as described above) occurs in some cases but not in all of them. Therefore, this was called geometry effects in VO-notched specimens subjected to mode I loading and these effects were studied for different geometry parameters (e.g., width/height ratio, notch opening angle, notch root radius, etc.). As its main novelty, the present study proved both experimentally and theoretically that the crack bifurcation can occur in VO-notch problems and, more importantly, the work predicted the values of both the bifurcation angle and its corresponding fracture load. Although the main object of the study was the fracture initiation in VO-notched specimens, we also studied two other cases (blunt V-notches and sharp V-notches) as two simplified examples of VO-notches with the aim of providing further validation. It is highlighted here that predicting the direction of crack initiation and the path of crack growth in the VO-notch problem can play an important role in estimating the level of damage imposed on the corresponding whole structure. Depending on the path of crack growth, the fractured VO-notched structure can experience either a total failure with significant economic costs, or alternatively a local material separation with insignificant damage. 

It is finally noted that the present manuscript investigated the fracture behavior of VO-notched specimens under static mode I loading. It would be very useful to extend the application of the same phenomenon to dynamic loading [32,33,34] and to study the dynamic fracture behavior of VO-notched specimens.

## 6. Conclusions

The geometry effects on the brittle fracture behavior of a number of CT and DCB specimens containing a VO-notch made of PMMA under mode I loading conditions have been studied through both experimental tests and theoretical analyses. It has been shown that although loading and geometrical conditions of all specimens were symmetric, for some specimens the fracture deviated from the notch bisector line. The effects of the changes in the geometrical parameters, such as specimen width, the notch depth, the notch opening angle and the notch radius, on the crack initiation angle and on the fracture load have also been assessed. For CT specimens under mode I loading, the crack growth path always remains along the notch bisector line. By increasing the width of the specimens (i.e., the DCB^1^ and DCB^2^ specimens), the crack initiation angle considerably differs from those observed in CT specimens. The crack initiation angle decreased in the VO-notched DCB specimens when either the specimen notch opening angle or the notch root radius increased. There was an enhancement in the fracture load of VO-notched DCB specimens for both larger notch root radii or for smaller notch opening angles and smaller widths. In addition, the effect of the blunt tip of V-notches on the fracture angle was checked by implementing some experimental mode I tests on a number of blunt V-notched DCB specimens made of PMMA. It was shown that by increasing the V-notch root radius from zero to a value just over zero, the crack initiation angle suddenly drops to zero and by additional augmentation of the notch root radius, the crack initiates in larger angles.

Furthermore, a stress-based fracture criterion, the generalized maximum tangential stress (GMTS) criterion, was derived by considering the first three terms of the stress field solution. The GMTS theory gives accurate predictions of the experimental crack nucleation angle and the fracture load for VO-notched specimens under pure mode I loading conditions.

## Figures and Tables

**Table 1:** Dimensions of CT, DCB^1^ and DCB^2^ specimens.

Specimen	a (mm)	W (mm)	h (mm)
CT	15	30	30
DCB^1^	45	90	30
DCB^2^	75	150	30

**Table 2:** Experimental fracture loads and fracture angle of the VO-notched specimens.

γ (°)	ρ (mm)	Specimen Type	Fracture Load (N)				Standard Deviation	Fracture Location (°)				Standard Deviation
			F1	F2	F3	F_average_		θ_0,1_	θ_0,2_	θ_0,3_	θ_0,average_	
15°	2.5	CT	111	155	148	138	19.30	0	0	0	0	0
		DCB^1^	67	59	51	59	6.53	74	63	72	70	4.78
		DCB^2^	43	48	59	50	6.68	76	75	71	74	2.16
	3.0	CT	155	154	147	152	3.55	0	0	0	0	0
		DCB^1^	75	79	65	73	5.88	68	70	69	69	0.82
		DCB^2^	52	49	43	48	3.74	73	76	69	73	2.87
30°	2.5	CT	144	124	110	126	13.59	0	0	0	0	0
		DCB^1^	79	68	84	77	6.68	73	65	76	71	4.64
		DCB^2^	45	53	46	48	3.55	76	71	71	73	2.36
	3.0	CT	166	145	151	154	8.83	0	0	0	0	0
		DCB^1^	79	86	84	83	2.94	65	69	64	66	2.16
		DCB^2^	43	40	46	43	2.45	71	68	72	70	1.70

**Table 3:** FEOD outputs for the VO-notched specimens under the unit reference load and mode I loading conditions.

γ (°)	ρ (mm)	Specimen Type	A_1_	A_2_	A_3_
15	0.125	CT	0.0749	0.0155	−0.0169
		DCB^1^	0.1336	0.0528	−0.0394
		DCB^2^	0.2069	0.0892	−0.0640
	2.5	CT	0.1029	0.0168	−0.0173
		DCB^1^	0.2620	0.0514	−0.0451
		DCB^2^	0.4142	0.0880	−0.0744
	3.0	CT	0.1107	0.0178	−0.0183
		DCB^1^	0.3122	0.0550	−0.0489
		DCB^2^	0.4961	0.0946	−0.0810
30	0.125	CT	0.0698	0.0161	−0.0177
		DCB^1^	0.1287	0.0507	−0.0398
		DCB^2^	0.2013	0.0794	−0.0638
	2.5	CT	0.1001	0.0171	−0.0176
		DCB^1^	0.1963	0.0602	−0.0494
		DCB^2^	0.3046	0.1017	−0.0725
	3.0	CT	0.1083	0.0181	−0.0206
		DCB^1^	0.2171	0.0653	−0.0552
		DCB^2^	0.3446	0.1114	−0.0916

**Table 4:** Comparison between the average experimental fracture angle and GMTS estimations.

γ (°)	ρ (mm)	Specimen Type	Fracture Angular Locationθ_0_ (°)		
			Experimental	GMTS	Deviation (%)
15	2.5	CT	0	0	0.00
		DCB^1^	70	75	7.14
		DCB^2^	74	80	8.10
	3.0	CT	0	0	0.00
		DCB^1^	69	78	13.04
		DCB^2^	73	79	8.21
30	2.5	CT	0	0	0.00
		DCB^1^	71	73	2.81
		DCB^2^	73	77	5.47
	3.0	CT	0	0	0.00
		DCB^1^	66	69	4.54
		DCB^2^	70	75	7.14

**Table 5:** Mode I loading test results of blunt V-notched specimens and VO-notched specimens.

γ (°)	ρ (mm)	Specimen Type	Experimental Fracture Load (N)		Fracture Angular Location(°)	
			V-Notch	VO-Notch	V-Notch	VO-Notch
15	2.5	CT	187	138	0	0
		DCB^1^	103	59	67	70
		DCB^2^	87	50	73	74
	3.0	CT	194	152	0	0
		DCB^1^	137	73	64	69
		DCB^2^	53	48	68	73
30	2.5	CT	174	126	0	0
		DCB^1^	98	77	69	71
		DCB^2^	67	48	70	73
	3.0	CT	207	154	0	0
		DCB^1^	102	83	63	66
		DCB^2^	63	43	66	70

**Table 6:** A comparison between the test results obtained from sharp V notched specimens (ρ = 0.0 mm) [17] and VO-notched specimens (ρ = 2.5 mm).

γ (°)	ρ (mm)	Specimen Type	Fracture Load (N)	Fracture Initiation Angle (°)
15	0.0	CT	90.3	0
		DCB^1^	45	43
		DCB^2^	30.5	46
	2.5	CT	138	0
		DCB^1^	59	70
		DCB^2^	50	74
30	0.0	CT	67.3	0
		DCB^1^	48.6	30
		DCB^2^	30.6	35
	2.5	CT	126	0
		DCB^1^	77	71
		DCB^2^	48	73

**Table 7:** Results of the fracture strength predicted through MTS and GMTS criteria.

γ (°)	ρ (mm)	Sample Type	Fracture Strength, $K_{If}^{VO}$ (MPa·mm^1−λ1^)		
			Exp.	Theoretical	
				MTS	GMTS
15	2.5	CT	87.25	87.23	87.23
		DCB^1^	65.68	87.22	65.87
		DCB^2^	63.04	87.20	62.96
	3.0	CT	94.21	94.16	94.15
		DCB^1^	70.04	94.14	69.99
		DCB^2^	67.99	94.14	67.95
30	2.5	CT	84.94	85.01	85.01
		DCB^1^	63.51	84.98	63.48
		DCB^2^	60.79	84.97	60.74
	3.0	CT	91.78	91.74	91.74
		DCB^1^	67.49	91.74	67.44
		DCB^2^	65.61	91.72	65.55

## Data Availability

The data presented in this study are available on request from the corresponding author.
